# What Do Parents Expect in the 21st Century? A Qualitative Analysis of Integrated Youth Care

**DOI:** 10.5334/ijic.5419

**Published:** 2020-08-17

**Authors:** Laura A. Nooteboom, Chris H. Z. Kuiper, Eva A. Mulder, Peter J. Roetman, Janna Eilander, Robert R. J. M. Vermeiren

**Affiliations:** 1 Department of Child and Adolescent Psychiatry, Curium-Leiden University Medical Centre, Leiden, NL; 2 Leiden University of Applied Sciences, Zernikedreef, Leiden, NL; 3 Horizon Youth Care and Special Education, Mozartlaan, Rotterdam, NL; 4 Academic Workplace Youth At Risk, Intermetzo-Pluryn, Nijmegen, NL; 5 Department of Child and Adolescent Psychiatry, Amsterdam University Medical Centre – Location VUMC, De Boelaan, Amsterdam, NL; 6 Youz: Parnassia Group, Dr. van Welylaan, The Hague, NL

**Keywords:** integrated care, families, parents, mental health, shared decision making

## Abstract

**Introduction:**

To provide integrated Youth Care responsive to the needs of families with multiple problems across life domains, it is essential to incorporate parental perspectives into clinical practice. The aim of this study is to advance our understanding of key components of integrated Youth Care from a parental perspective.

**Methods:**

Semi-structured interviews were administered to 21 parents of children receiving Youth Care from integrated care teams in the Netherlands. Qualitative content analysis was conducted by means of a grounded theory approach following qualitative reporting guidelines.

**Results and discussion:**

Parental perspectives were clustered into six key components: a holistic, family-centred approach; addressing a broad range of needs in a timely manner; shared decision making; interprofessional collaboration; referral; and privacy. Parents emphasized the importance of a tailored, family-centred approach, addressing needs across several life domains, and active participation in their own care process. However, they simultaneously had somewhat opposing expectations regarding these key components, for example, concerning the changing roles of professionals and parents in shared decision making and the value of involving family members in a care process. Professionals should be aware of these opposing expectations by explicitly discussing mutual expectations and changing roles in decision making during a care process. To enable parents to make their own decisions, professionals should transparently propose different options for support guided by an up-to-date care plan.

## Introduction

Sustainable change in Youth Care can only be achieved in cooperation with all parties involved, especially parents and their children [1]. Previous studies have shown that client perspectives demonstrate low convergence with quality indicators based on clinicans, research, and policy [2,3]. Clients often value functional outcomes in the context of everyday living and quality of life over control of their illness [4,5]. Moreover, incorporating client perspectives into clinical practice is associated with improved working alliance, increased satisfaction with services and autonomy support [3]. Thus, to provide integrated Youth Care responsive to the needs of families, it is essential to incorporate parental perspectives into clinical practice [6]. Therefore, this study aims to advance our understanding of key components of integrated Youth Care from a parental perspective.

Youth Care encompasses the support for children aged 0–23 years and their families who need support from a variety of services, including preventive health services, youth mental health support, and specialized (mental health) care [7]. Families in Youth Care with multiple needs often deal with a plurality of (enduring) co-occurring psychosocial problems in various areas of life [8]. It is difficult to support these families due to the interactions between problems, the varying needs of families, the organization of care focusing on single needs, and a lack of coordination between the multiple care services involved [8,9,10]. If left untreated, these problems adversely affect a child’s development and family functioning, leading to an increased burden on social, familial, and academic functioning that tend to persist into adulthood [11]. For example, unsupported mental health problems can eventually lead to social isolation, poor educational achievement, emotional dysregulation, and parental distress [12,13].

To meet the needs of these families, Youth Care professionals seek to promote coherent, continuous, and coordinated care across several life domains, also defined as integrated Youth Care [8,14]. The aim of integrated Youth Care is to coordinate services around families’ needs and improve quality of support by incorporating services, and ensuring collaboration between professionals [14]. Providing this integrated Youth Care has increasingly been recognized as a necessity by professionals, policy makers, researchers, youth, and parents as it can be effective to improve the care process and families’ satisfaction with care [5,14,15,16]. Altough evidence on the effectiveness of integrated Youth Care is promising [17,18], there is a gap between empirical support for the effectiveness of integrated approaches and the efficacy of these models in actual practice [19].

Following the principles of Evidence Based Practice to organize high-quality care, it is crucial to combine client perspectives, clinical experiences, and evidence from research [20]. As previous research to youth engagement in the organization and policy of services suggested, it is important to engage children and their families in developing integrated care, since this can increase service uptake, engagement in-, and control over their care process, and satisfaction over services [21,22,23,24]. These studies recommend to organize accessible and welcoming locations with minimal waiting times, where youth feel valued and respected. Also, co-location of services, offering walk-in sessions, and meeting youth at a location of their choice can increase accessibility [21,24]. However, these recommendations cannot be generalized to parental perspectives, since youth perspectives do not necessarily align with those of parents.

Moreover, a limited number of studies have attempted to determine key components to integrated Youth Care from a parental perspective. For example, a small qualitative study focusing on a specific population (parents of children with anxiety and depression), demonstrated that the presence of a care coordinator enabled parents to focus on their child, instead of coordinating the care process among multiple professionals [25]. Also, this study found that integrated Youth Care was hindered by a lack of clarity with respect to allocation of responsibilities and confidentiality issues between professionals [25]. Studies on integrated care in other fields of interest, for example, general health care and adult services, have found that from a client perspective, timely access to services, smooth transitions between health care providers, adequate exchange of information, and co-location of services are important aspects of integrated care [14,19]. However, integrated care has been deemed a highly context-dependent process and there is no single example or best practice applicable to all settings [24,26,27].

An important issue in integrated Youth Care is determining the focus of support. One of the leading principles of decision making in integrated Youth Care is shared decision making, in which clients and professionals collaborate to make decisions about a care process [28,29]. Although shared decision making can lead to improved client satisfaction and self-support skills, implementing shared decision making across settings is intricate [28]. Particularly in integrated Youth Care, shared decision making can be complicated by difficulties in prioritization of needs, sequencing of services, conflicting needs of family members, and a large number of professionals involved in a care process [28,30,31]. Previous research demonstrated that parents and youth might need support for their role in decision making [32]. Moreover, Youth Care professionals often experience difficulties incorporating multiple perspectives into a comprehensive care plan [5,33]. Disagreement between youth, parents, and professionals concerning the form and intensity of support also hinder shared decision making [20,30].

Yet, despite the importance of incorporating client perspectives into clinical practice, little attention has been paid to parental perspectives on integrated Youth Care and decision making processes [2]. Parents are crucial in children and young persons’ lifes and their recovery process [34]. Also, they are, especially in young children, the first point of contact with professionals and play an important role in treatment participation [35]. Therefore, this qualitative study sets two objectives: (1) to identify what parents consider key components of integrated care, and (2) to describe facilitators and barriers of integrated Youth Care according to parents. The objectives are to advance the understanding of integrated Youth Care from a parental perspective, to eventually enable professionals to tailor integrated Youth Care to the needs of families with multiple needs Double. Also, the results of this study might encourage services and policy makers to include quality indicators that reflect integrated Youth Care from a parental perspective.

## Methods

### Setting

This study is part of the research project of the Academic Workplace ‘Gezin aan Zet’ (translated: Family‘s Turn), a collaborative initiative in the Netherlands, involving stakeholders from practice (youth and parents, professionals), academia, policy, and education. The current study focusses on parents receiving support from full-integrated, multidisciplinary care teams, the so-called Youth Teams that operate in almost all municipalities in the Netherlands (see also **Text Box 1** for the context of Youth Teams) [36,37]. Each Youth Team consists of eight to twelve professionals with different expertise (e.g., social work and education, specialized mental health care, infant mental health, (mild) mental retardation, coaching, parenting support, and child protection; [38]). Youth Teams support families as much as possible within their own environment and operate within a primary care setting as a linking pin between universal services and specialized care [7]. If necessary, they provide short-term, ambulatory support or refer to more specialized Youth Care, following a matched- or stepped-care approach [39,40]. In total, six Youth Teams from two regions in the Netherlands (Holland Rijnland and The Hague) participated in the overal research project.

**Text Box 1: The context of Youth Teams**

In 2015, there has been a decentralization of the Youth Care system in the Netherlands. Currently, municipalities are responsible to organize and provide Youth Care on a local level, including preventive support, primary care, specialized mental health care, and child protection. Arguments for this decentralization were reported deficiencies concerning an increased use of care, pressure on specialized care, fragmentation of support, and a lack of interprofessional collaboration. Municipalities aim to provide accessible, integrated care within families’ own environment by decompartmentalization of budgets and organizing local support for children and their families with a variety of psychosocial, stress-related, and socio-economic needs.

In almost all municipalities in the Netherlands, multidisciplinary care teams (i.e. Youth Teams) are operative to organize and provide integrated Youth Care on a local level. All professionals in Youth Teams have a broad range of tasks to ensure high quality support for children and their families, with a focus on empowerment, strengthening the capacities of families, involving the social network of families, and provide early detection and support. Although specific tasks and team composition of a Youth Team varies depending on local needs, professionals generally have four major functions: (i) accessible support by means of consultation, advice and basic diagnostics to identify needs, (ii) a linking pin between universal services and specialized Youth Care, (iii) coordinate support in collaboration with other (local) services, and (iv) provide ambulatory support if needed.

Since professionals in Youth Teams provide support to families with a broad variety of needs, they operate with a generalist view on the entire family’s welfare, and a specialist focus on specific needs (for example specialized mental health care, parental support, child protection). Professionals in Youth Teams are responsible to preserve their specialism by means of training and supervision. Due to its multidiscplinary character, Youth Teams can provide a broad range of services, leading to increased access of support. Also, professionals can learn from each other’s expertise, by closely collaborating in care processes. To improve collaboration, professionals in Youth Teams meet every week to discuss cases and team functioning. Moreover, as a linking pin between universal services and specialized mental health care, Youth Teams closely collaborate with local general practitioners, schools, services for adult mental health support, financial support, and preventive services.

### Participants

Parents were invited to participate in a semi-structured interview by an email from their Youth Team professional. As we aimed to prevent convenience sampling bias, professionals were encouraged to approach all parents in their caseload [41]. The email contained a description of the project and the process of interviewing (audio-taping, confidentiality, and the right to withdraw at any moment). A parent representative (BH) supported the researchers in formulating comprehensible content to ensure that parents understood the information. After parents expressed their interest, they were called by a student of the Leiden University of Applied Sciences, who worked under the supervision of the researchers (LAN and JE). From the 22 parents who were approached, one parent refused to participate due to a lack of time.

To guarantee parental perspectives were based on actual experiences, we purposively included parents who had at least three meetings with a Youth Team professional [42]. There were no further criteria for in- or exclusion, since we aimed to involve a heterogeneous group of parents, representing the diverse population of families in Youth Care. After parents agreed to participate, the interview was scheduled at a place of their choice, mostly at home. All parents gave written informed consent prior to the interview. The researchers had no prior knowledge of the participants and vice versa. All parents, except one mother, filled in a demographic survey, her data was listed as missing. The Medical Ethics Review Board of Leiden University Medical Centre concluded that the research project was not subject to the Medical Research Involving Human Subject Act (WMO) and complied with the Netherlands Code of Conduct for Research Integrity. The Consolidated criteria for Reporting Qualitative Research [43] were applied to promote transparency and ensure clear and comprehensive reporting of the study methods (Appendix A).

### Data collection

To shed light on the complex process of integrated care and allow parents to express their experiences, semi-structed interviews were conducted [31]. A topic list with open-ended questions was formulated in advance based on previous studies of client perspectives on integrated care [19,25]. The topic list was supplemented with input from a reflexive meeting of the authors (LAN, JE, EAM, CHZK) and two Youth Team professionals. Subsequently, the topic list was pilot tested on a parent representative (BH) and minor linguistic adjustments were made. Next to general questions on the support of a Youth Team and overall satisfaction, the topic list included questions on: (i) a family-centred focus (e.g. experiences with the involvement of family members and the social network in a care process), (ii) collaboration between professionals and parents (e.g. attitudes and communication of professionals towards parents), (iii) parental involvement in shared decision making (e.g. how parents experienced their roles in decision making processes, experienced freedom to adapt treatment plans), (iv) interprofessional collaboration and joint meetings (e.g. parental experiences with joint meetings and collaboration between professionals involved in the care process), (v) experiences with a shared care plan (e.g. whether there was a care plan, and the role parents played in formulating the care plan), (vi) availability of care (e.g. time between application for support and first meeting, availability of specific support), and (vii) privacy-issues (e.g. confidentiality of information and communication between professionals).

The interviews were conducted between February and June 2017 by two students (one male and one female) of the Leiden University of Applied Sciences, accompanied by a researcher experienced in interviewing and qualitative data analysis (LAN or JE, both female). Field notes were obtained during each interview. A reflexive meeting to evaluate the interview process and discuss new insights between the student and one of the researchers (LAN or JE) took place after each interview. All parents were assigned a study number to guarantee anonymity. Each interview was audio-recorded and transcribed (verbatim) afterwards. Parents were asked if they wanted to comment on the transcripts, however, no parent was interested in doing so. The presented quotes have been translated from Dutch to English by three researchers (LAN, SvdD, PJR). Due to the verbatim transcription, the quotes presented in our results section contain literal wordings and therefore, might not be completely fluent.

### Analysis

All transcripts were imported into ATLAS.ti (version 7), a computer program for labelling and organizing text content. In analysing the transcripts, we applied a triangulation approach by using both inductive and deductive strategies [44]. A coding tree was developed and applied based on the topic list, supplemented with codes that arose from open coding based on the grounded theory approach [45]. Two researchers (LAN and JE) discussed each coded transcript to resolve differences in coding. No additional codes were added after coding approximately 15 out of the 21 interviews, an indication that saturation was reached and no supplemental interviews were needed [46]. Second, axial coding took place by further analysis and merger of the coded fragments, resulting in six key components [47]. During reflexive meetings, the researchers (LAN, JE, EM) and parent representatives (BH and CdK) discussed the interpretation of the codes and components. Subsequently, the first author (LN) deductively compared the themes that emerged from the thematic analysis by re-reading the transcripts. By applying this bracketing method, we have limited possible adverse effects of prejudices that may have affected the research process [48].

## Results

### Demographics

In total, 21 parents were interviewed, 17 mothers and 4 fathers, all from different families. The interview duration ranged from 31 to 92 minutes (M = 53 minutes). Eleven parents provided information regarding their child’s age, that ranged from 3 to 21 years (n = 17, M = 11.23). Although the diagnosis of the child and type of familial problems were not explicitly asked, all parents had received support from professionals of Youth Teams, supplemented with other services such as specialized mental health-, mediation-, or financial support services. This is an indication of multiple needs across several life domains. For an overview of demographic characteristics of the parents, see Table 1.

**Table 1:** Demographic characteristics of the parents.

Variable	

Gender	
Male [n(%)]	4 (19.1%)
Female [n(%)]	17 (80.9%)
Age in years	
Mean age in years (SD)	43.75 (8.47)
Age range in Years	26–57
Cultural Background	
Western [n(%)]	17 (85.0%)
Non-Western [n(%)]	3 (15.0%)
Highest Educational Level	
Primary Education [n(%)]	2 (10.0%)
Intermediate Vocational Education [n(%)]	8 (40.0%)
Higher Vocational Education [n(%)]	7 (35.0%)
University [n(%)]	3 (15.0%)
Marital Status	
Two-parent household [n(%)]	10 (50.0%)
Divorced [n(%)]	9 (45.0%)
Single-parent household [n(%)]	1 (5.0%)
Total Number of Children	
One child [n(%)]	5 (25.0%)
Two or more children [n(%)]	15 (75.0%)

*Note*: N = 21.

### Findings

Parents described integrated Youth Care as a process where multiple professionals collaborate to provide adequate care for the entire family. Overall, parents were satisfied with the support of local multidisciplinary Youth Teams. Despite the heterogeneity of the participants, our results show a high consensus between parents in their perspectives on integrated Youth Care. Based on the open coding of the interviews, six key components were formed, displayed in Table 2 and described in Appendix B. These components will be further explained in the following section.

**Table 2:** Components of integrated Youth Care according to parents.

Component	Description

Holistic, family-centred approach	A holistic approach of needs and strengths of all family members.
Addressing a broad range of needs in a timely manner	Timely support across several life domains, tailored to a family’s needs.
Shared decision making	Parental involvement in decision making processes.
Interprofessional collaboration	Collaboration between professionals with different expertise, or from different organizations.
Referral	Transition from one care provider/organization to another.
Privacy	Privacy of family members during information exchange.

#### Holistic, family-centred approach

All parents emphasized the importance of a holistic, family-centred approach in integrated Youth Care: a focus on a families’ welfare across several life domains, instead of solely addressing the needs of the child with the most explicit problem behaviour. Parents’ main argument for a family-centred approach was that the problems of one family member often influence the entire family’s well-being. Addressing the welfare of all family members, was experienced as having a positive effect on a family’s capacity to addressing their needs.

“When a single person has a problem, this in turn also has its effect on the rest of the family. So, it is great to start together in the assessment phase, and to continue individually during the care process.”

Parent 3.1

To facilitate a complete overview of families’ functioning, various parents described that professionals should incorporate all family members’ perspectives on needs and strengths, supplemented by the perspectives of teachers and other professionals like general practitioners. According to most parents, discussing the various perspectives with families led to new insights into needs and strengths, which in turn resulted in a feeling of empowerment and positively influenced the care process.

”And I can tell my story, but I see it from one direction. I want an extra pair of eyes that look at the situation from different angles. In the end, that went very well, because of the open communication with school and the general practitioner.”

Parent 1.1

A barrier in mapping the entire families functioning was that some parents experienced uneasiness the moment a professional asked questions about family functioning across several life domains, without explicitly mentioning the importance of asking these questions. To illustrate, one parent was confused that a professional asked about her/his family’s financial situation, while the initial application for support was based on a child’s externalizing behavioural problems at school.

“The reason why they actually want to know so much about us, while I only asked a question about my son or daughter. And when an explanation is given, then you think ‘all right, on the one hand it makes sense, so it’s a plan for the whole family, the functioning of the whole family’. I do understand that.”

Parent 2.1

Alongside the family-centred approach, Youth Team professionals often proposed to involve a family’s personal social network for informal support. By drawing a visual overview of the social network, parents reported that they gained more insight in the people whom they can cask for informal support. A facilitator in involving the social network was that parents chose by themselves who they approached, this was not dictated by professionals. Some parents experienced that involving grandparents, friends or neighbours as support resulted in more energy and strength to face problems. Importantly, not all parents felt the need to involve their personal social network in the care process. Barriers in involving the social network were cultural and generational differences in talking about problems and a social network that was already overburdened.

“My mother is from a different generation, and she says: ‘these kinds of problems you have to solve yourself, do not air your dirty laundry’.”

Parent 1.7

#### Addressing a broad range of needs in a timely manner

In integrated Youth Care, addressing the needs of all family members in a timely manner was reported as essential. However, parents emphasized that an integrated approach does not mean that all needs should be addressed simultaneously. In fact, too many treatment goals at the same time resulted in overburdening of families, hindering the care process. Jointly prioritize needs and decide on the focus of support was described as a facilitator, while focusing on a family’s needs instead of a supply-oriented approach.

“I like the fact that not everyone is placed inside a box of ‘that is how you function, and we are going to solve it in the following standard ways.’ No, they really assessed our individual needs.”

Parent 3.2

All parents reported long waiting lists, often for specialized services as a major barrier to addressing needs in a timely manner, leading to insufficient support, stagnation of the care process, an increase in needs and difficulties in interprofessional collaboration. Nevertheless, parents differed greatly in their perceptions of waiting times. This variety seemed related to the severity of problems: the more severe the problems, the more urgent the need for help, and the longer the perceived waiting time. Furthermore, a lack of clarity of services and specific demands of organizations (e.g. refusing family members with comorbid problems) were described as barriers in integrated Youth Care. Parents emphasized the need of transparent communication about waiting times and the type of services offered by organizations.

“Because [organization] is not always clear in what they can provide and can’t provide, the Youth Team cannot adapt to this. So, the communication and the care offered were not always clear. So sometimes it is not entirely clear what one party does and what the other party does. And the communication is just rigid, making it very difficult to coordinate things.”

Parent 4.2

#### Shared decision making

Multiple examples of shared decision making in integrated Youth Care were described in the interviews: the need for jointly assessing priorities during the care process, the value of making their own decisions on the type and intensity of care, and the increased motivation parents experienced due to the involvement during all stages of care. Freedom of choice and transparent communication about different options for support were reported as facilitators by parents to make their own decisions.

“No, the decisions are coming from me and my husband. But the coach gave us advice, just for the decision. But we made the decision. We can accept these advices, but we also can just say no.”

Parent 2.2

An up-to-date care plan, shared with families and professionals promoted a transparent overview of the care process and gave insight in current and future goals and actions, facilitating shared decision making and leading to an increased consensus on the focus of support. Generally, a professional took the lead in formulating the care plan, by inventorying families’ needs and formulating goals. Importantly, parents expressed that they should always participate in this process, by formulating their own goals or adjusting the goals formulated by a professional.

“They gave us the feeling of being heard, leading to feelings of security, safety, and positivity, and increasing feelings that you can work on something.”

Parent 1.3

Frequent evaluation is necessary to maintain an up-to-date and flexible care plan, which is responsive to the changing needs of families. These evaluations should be initiated by professionals, and parents thought it is a professional’s responsibility to keep the care plan up to date. Some parents explicitly mentioned an increased feeling of involvement in the care process when developing or evaluating a care plan in collaboration with a Youth Team professional.

There were also barriers in shared decision making reported by parents. First, differences in the local organization of Youth Care, for example, between two adjacent regions, led to perceived disparities in access to services, and most importantly a perceived limited freedom of choice. Second, different views on adequate support, for example between professionals and parents, were experienced as having a negative effect on shared decision making. These differences were particularly problematic, since parents trust and value a professionals’ expertise, but on the contrary, they are experts on their own family situation. Third, in some cases the perceptions of the most appropriate support for families differed between various professionals involved, leading to confusion for parents. In case of differences in perceptions or a perceived limited freedom of choice, a parent suggested that professionals should transparently discuss all options with families.

“Professionals stated that he was better off at [organization]. I said, ‘yes but that is an organization you have a contract with, that is cheaper for you, but not appropriate for my son’. And then you get into a conflict (…). What I found most painful was that they did not look at my son’s needs, but what was financially appropriate for them.”

Parent, 3.2

#### Interprofessional collaboration

Beside the support of a Youth Team, all parents also received support from professionals of other services, like specialized mental health care centres or financial support services. Although many parents preferred support from one single professional or organization, they understood the importance of interprofessional collaboration to provide a broader range of support. Specifically, schools and general practitioners were mentioned as important collaborative partners, since they have known families for a longer period and are involved in their daily lives.

Multiple examples of facilitators and barriers in interprofessional collaboration were reported. For example, familiarity between professionals, frequent communication, and accessibility of professionals were mentioned as facilitators. Also, parents emphasized the importance of clear allocation of tasks and responsibilities, especially when there were multiple family members and professionals involved. Interprofessional collaboration, by ensuring clear communication and coordinated support, should be initiated by professionals, but always with parental consent.

“I think we were heard, but I think the problem is just the structure. There is just not one person with the final responsibility within the specialized mental health care, who consults our coach. There were all super competent people, but one is about diagnostics, the other one about autism treatment, the other is the psychiatrist…. But there is not one person who says: ‘I will take the lead’.”

Parent 3.2

Co-location of services, multidisciplinary care meetings, and a care coordinator were forms of interprofessional collaboration described by parents. Co-location was experienced to have a positive effect on the accessibility of care, by reducing the threshold of seeking help for a broad range of problems. Furthermore, parents experienced that co-located professionals were more familiar with the other professionals’ services, leading to increased interprofessional communication, reduced fragmentation of services, and early support. Overall, parents reported to be more satisfied with interprofessional collaboration between professionals from one Youth Team compared to collaboration between professionals from different organizations. Due to the multidisciplinary organization of Youth Teams, parents felt that diverse expertise was easily accessible, increasing the efficiency of the care process. Moreover, parents experienced that Youth Teams had short lines of communication with universal services like schools, general practitioners, and child healthcare centres in the neighbourhood. For example, Youth Team professionals were frequently co-located at visible locations, like schools or police stations, leading to an increased accessibility of care and early support.

All interviewed parents had participated in multidisciplinary care meetings. During these meetings, the care process was discussed among the family, the professionals involved, and sometimes the personal social network of the family. Although parents described these meetings as valuable to create an overview of the care process and to reduce fragmentation in support, parents stressed that the meetings were sometimes burdensome. Sufficient preparation facilitated multidisciplinary meetings, both for professionals and the parents, for example by formulating an agenda beforehand. Moreover, parents found it essential that professionals adjusted their pace and language during multidisciplinary meetings, and that there was someone (a professional or someone from a family’s network) available to support parents expressing their needs.

“And also, in response to large meetings, where 17 people were sitting around the table. I felt so alone. There were 17 people around the table and I needed someone to stand by me, who, together with me, stood up for my child.”

Parent 6.2

A reported barrier in organizing multidisciplinary meetings was the lack of availability of professionals. Some parents noticed that it was not always a necessity to organize or participate in a face-to-face meeting to come to an agreement. Discussions by phone or email would also have been sufficient and easier to organize, as long as there is transparent reporting to parents afterwards.

A care coordinator, described as a professional with the formal task to maintain an overview of the care process and to stimulate interprofessional collaboration, was reported as an important facilitator to interprofessional collaboration. In fact, a lack of care coordination led to fragmentation of support, a major barrier in integrated Youth Care. Another reported barrier was the high turnover rate of professionals. Due to this turnover rate, parents had to tell their stories repeatedly and form relationships with several professionals, leading to resistance and overburdening of families. Also, the changing composition of a care team led to indistinct responsibilities and a lack of communication between professionals.

“It would have been great if there was just one professional that supported our family.”

Parent 1.5

#### Referral

Many parents were referred from one organization to another, mostly from local Youth Teams to more specialized mental health care services. To facilitate the referral process professional should have knowledge of local services and the skills to efficiently identify the needs of families. During referral, parents were often requested to provide personal information. Although most parents understood the importance of sharing this information, some felt uncomfortable sharing personal information with unfamiliar professionals or organizations. Warm handoffs were mentioned as facilitating the referral process, described as the transition from one care provider to another, in which a professional supported parents with sharing relevant information. Parents often had to wait for available support, a barrier in the referral process. During this transition phase, it is essential that there is a contact person for questions and if necessary, a minimum of support available.

“The professional continued to support [me] until the care was handed over, which was very nice. She joined us to the consultation where the diagnosis and treatment were discussed with the psychiatrist. And she says, you know, if you’d like, I could come along. I could coordinate what [organization] will do and what I’ll do.”

Parent 4.5

#### Privacy

Parents emphasized two elements of privacy that were of importance during an integrated care process. First, professionals should consider the privacy of all family members. Specifically, professionals cannot presume that all family members involved in a care process can receive all information reported by other family members. For example, during meetings with the entire family, caution is needed when sharing information that was discussed in previous, individual support sessions. A reported strategy to ensure the privacy of all family members was a discussion of the information that can be shared with other family members beforehand.

According to parents, the second element of privacy was the exchange of information between professionals. All parents understood the importance of information exchange between professionals to adjust support. However, a barrier to integrated care was that professionals sometimes exchanged information without parental consent. This led to distrust and confidentiality issues, negatively influencing the integrated care process. To facilitate information exchange, professionals should always explain the importance and content of the information that will be shared and explicitly ask for permission to do so.

“The professional did not go behind my back to call my daughters school and inform on how she was doing. No, she did not do that and that was good. In advance, she asked whether I had any problems with her going to my daughter’s school.”

Parent 1.2

## Discussion

Thus, what do parents expect from integrated Youth Care in the 21st century? In this qualitative study we identified six key components of integrated Youth Care according to parents: (1) a holistic, family-centred approach, (2) addressing a broad range of needs in a timely manner, (3) shared decision making, (4) interprofessional collaboration, (5) referral, and (6) privacy. Parents described several facilitators, including: transparent communication, involvement in the care process, freedom of choice, comprehensive and up-to-date shared care plans, and clear allocation of tasks and responsibilities between professionals. Unfortunately, a perceived lack of access to services, long waiting lists and difficulties in interprofessional collaboration hindered integrated Youth Care. When comparing these results to previous findings from studies on integrated care from the perspective of youth, we conclude that there are similarities in themes identified [21,22,23,24]. Both parents and youth stressed the importance of accessible support with minimal waiting times, co-location of services, and engagement in decision making.

In this study, we explicitly studied parental perspectives. Parents stressed the importance of addressing a broad range of needs across several life domains. However, an integrated approach does not mean that all needs should be addressed simultaneously since this can lead to overburdening of families. Parents value a tailored, family-centred approach, which addresses needs across several life domains and requires active participation in a care process of both parents and professionals. However, they also held somewhat opposing expectations regarding these key components. In the following section we reflect on our findings and provide implications for practice, policy, and future research.

A holistic, family-centred focus was the first component of integrated Youth Care, which focusses on the welfare of the entire family across several life domains. Confirming previous research, parents emphasized that a family-centred approach strengthened a family’s capacity to identify and address needs, leading to increased feelings of empowerment, ownership of, and involvement in a care process [49]. Professionals should explicitly stress the importance of a holistic, family-centred approach, since some parents experienced uneasiness and confusion during broad assessment of all family members on several life domains. Furthermore, although some parents valued the involvement of their personal social network in the care process, there were also parents who did not want to involve their network, especially when they considered their network as overburdened. This is problematic, since families with multiple needs are a population from which we expect to benefit most from a supportive, informal social network [50]. There is a need for increased efforts of Youth Care professionals to organize informal support for these families, for example by introducing peers or experienced experts as support [51]. Including these experienced experts in integrated care has also been identified as a facilitator in previous research to integrated care from youth perspectives [23].

A major barrier in addressing a broad range of needs in a timely manner, the second key component of integrated Youth Care according to parents, was a lack of access and availability of services. According to parents, this was due to long waiting times and a lack of clarity concerning the type of services offered by organizations. A lack of access and availability negatively influences the care processes, for example by lowering attendance for appointments [21,22,23,24,25,26,27,28,29,30,31,32,33,34,35,36,37,38,39,40,41,42,43,44,45,46,47,48,49,50,51,52]. Moreover, parental perceptions of waiting times differed greatly by severity: the more severe the problem, the more urgent the need for support and the longer the perception of the waiting time. In line with previous research on youth perspectives [21], parents emphasized that transparent communication about availability of services positively influenced the perceived waiting time. This in turn had a positive effect on the care process, since parental expectations were more aligned with the actual situation. In improving transparency of availability of services, future research should focus on creating innovative (digital) systems with up-to-date information on the availability of services.

Regarding shared decision making, the third component of integrated Youth Care, most parents highlighted the importance of making their own decisions about the type and intensity of care. Multiple parents suggested that the brunt of the responsibility in shared decision making should be with families, and that a professional’s main task is to inform parents about the options for support. This finding seems somewhat contradictory to the principles of shared decision making, namely that professionals and families share responsibility over a care process, discuss multiple options for support, and make joint decisions [28,53]. The focus on the word ‘own’ seems in line with the worldwide trend of growing participation of clients in health care decisions and health consumerism, in which clients have increased responsibility in their own care trajectories, but also place high demands on immediate, personalized services [54].

Particularly when perspectives on the most appropriate focus of support differ between parents, youth, and professionals, it is unclear who decides in shared decision making. Is a professional with expertise on child development and sequencing of services most suited to make a final decision, or the family, as an expert on their own situation? A complicating factor is that the extent of a family’s involvement in shared decision-making changes over time and often gradually develops during a care process [30]. This finding implicates that during a care process, responsibility for choices might shift from professionals to families. A possible explanation for these changing roles in shared decision making that we can draw from our study, is that families gain more insight in their needs and strengths during a care process, leading to increased feelings of empowerment, ownership and involvement in decision making processes. Although we did not explicitly ask for the roles of youth in decision making, it might be possible that decision-making power shifts from parents and professionals to children and youth as they grow older [55]. In line with previous research [32], we advocate that professionals must be aware of changing roles of families in shared decision making and discuss these roles over time. In that, professionals must consider (cognitive) capabilities of families, the age of children, and always discuss families values and preferences [56]. Unfortunately, to date there are few guidelines applied by professionals to discuss multiple perspectives and preferences in integrated care [5]. In our study, we found three major facilitators in shared decision making according to parents: (1) transparent communication, (2) an up-to-date care plan including an overview of the care process and goals for support, and (3) frequent evaluation of this care plan. Future research is warranted to further examine the roles and responsibilities of parents, professionals, and youth in shared decision making. In that, we recommend to consider eventual differences between parents and youth in their perspectives on the roles of children and youth in decision making processes, and under which conditions it is justified to disengage a professional, parent, or youngster from a decision-making process.

Concerning interprofessional collaboration, the fourth key component of integrated Youth Care, parents emphasized the importance of collaboration between schools and care professionals. However, collaboration between the two systems is fragmented due to differences in culture and language, but also in policy, roles, and tasks [57]. Since this collaboration is of such an indisputable importance for families in Youth Care, we strongly recommend professionals and policy makers to invest in collaborative care initiatives, focused on improving familiarity and communication between Youth Care professionals and schools.

A barrier regarding referral, the fifth key component of integrated Youth Care, was that due to turnover of professionals, parents had to tell their stories repeatedly, leading to resistance and overburdening of families. Previous research stressed that many transitions to other care professionals harm a care process, since it leads to difficulties in forming trusting relationships and reduces the likelihood of appropriate support being sought by the parents [58]. In line with previous research [25], parents from our study emphasized the importance to have a professional available for questions and, if necessary, to support transitions between organizations. This can be a professional in the role of a care coordinator, who supports a family during the entire care process and stimulates interprofessional collaboration. Future research should pay attention to the function of a care coordinator (e.g. psychologist, general practitioner, social worker) and its role, for example whether this coordinator should also provide ambulatory support directly to the family.

The sixth key component was the importance of privacy, both within families and between professionals. This component is strongly linked to the other key components, such as a family-centred approach, interprofessional collaboration, and referral. According to parents, professionals should always explain the importance of sharing information, and discuss beforehand what information will be shared with other family members or other professionals.

When reflecting on the setting we studied, we conclude that overall, parents were positive about the support from local, multidisciplinary Youth Teams, especially regarding interprofessional collaboration within a Youth Team. Furthermore, Youth Team professionals were visible in the neighbourhood because of co-location in schools and health care centres, leading to increased accessibility and early support. In line with previous research, we state that centrally and co-located services that facilitate accessibility of integrated support are preferable [21,23]. On the other hand, parents also mentioned several disadvantages of organizing Youth Care on a local level (e.g. local differences in organization of care, long waiting lists, and limited access of specialized services). Since measuring the effectiveness and efficiency of Youth Teams was beyond the scope of this study, we cannot conclude whether forming full-integrated teams on a local level is the most efficient way to provide integrated Youth Care. Future studies should focus on the type of services and expertise needed on a local level to effectively meet the needs of families with multiple needs across several life domains.

Our findings should be interpreted in the light of several strengths and limitations. By applying the Consolidated criteria for Reporting Qualitative Research [43], we promoted transparency and ensured comprehensive reporting of our study. A unique aspect of this study was the continuous and intensive involvement of parent representatives. The reflexive meetings with both parents and researchers limited potential negative effects of prejudice and helped the researchers to approach parents in an understandable way. We deliberately chose semi-structured interviews as our research method, to shed light on the complex process of integrated Youth Care and to allow parents to express their viewpoints [31]. However, a mixed-methods approach would also have been valuable to measure to what extent the key components influenced the actual care process [59]. Although we aimed to prevent convenience sampling bias, all parents we spoke to had generally positive experiences with the support from a Youth Team. For future studies it might be interesting to compare parents with positive and negative experiences with integrated support, to see whether there are characteristics that predict successful treatment outcomes and satisfaction with support. Furthermore, the relatively small number of participants and lack of geographic spread across the country might have negatively influenced the transferability of results to other contexts or situations [60]. Moreover, we lack specific information regarding the childrens’ age, type of needs, and intensity of support that families received. It would have been interesting to combine this specific information with the parental perspectives, perspectives of youth, and perspectives of the professionals involved, to study whether these components influence effectiveness and perspectives on integrated care. Also, for this study we included parents based on the assumption that most Youth Team professionals are in contact with the biological parents of children in care. In future studies, perspectives of alternate caregivers and other family members can be investigated further, since they might have other perspectives.

## Conclusion

The parental perspectives on integrated Youth Care in this study emphasize that parents have a strong desire for a family-centred approach and active participation in decision making over their own care process. However, since parental expectations regarding these key components of integrated Youth Care are somewhat opposing, professionals should be aware of potential confusion and explicitly discuss mutual expectations during a care process. Furthermore, since parental involvement in shared decision making is not fixed, professionals should frequently evaluate family’s roles and responsibilities with the help of an up-to-date care plan and transparently propose different options for support. There is a need for guidelines on how to discuss and decide in integrated care, specifically when there are multiple conflicting perspectives and preferences. Despite the organization of integrated care in local Youth Teams, parents still perceive a lack of access, long waiting lists, and difficulties in interprofessional collaboration. Therefore, it is crucial that both professionals and policy makers invest in collaborative care initiatives, for example between schools and Youth Care. Also, innovative ways to organize integrated Youth Care on a local level for families with multiple needs should be explored further.

## Additional Files

The additional files for this article can be found as follows:

10.5334/ijic.5419.s1 APPENDIX A. COREQ Checklist.

10.5334/ijic.5419.s2 APPENDIX B. Components and code scheme.
