# Correction: What Do Parents Expect in the 21st Century? A Qualitative Analysis of Integrated Youth Care

**DOI:** 10.5334/ijic.5613

**Published:** 2020-10-01

**Authors:** Laura A. Nooteboom, Chris H. Z. Kuiper, Eva Mulder, Peter J. Roetman, Janna Eilander, Robert R. J. M. Vermeiren

**Affiliations:** 1 Department of Child and Adolescent Psychiatry, Curium-Leiden University Medical Centre, Leiden, NL; 2 Leiden University of Applied Sciences, Zernikedreef, Leiden, NL; 3 Horizon Youth Care and Special Education, Mozartlaan, Rotterdam, NL; 4 Academic Workplace Youth At Risk, Intermetzo-Pluryn, Nijmegen, NL; 5 Department of Child and Adolescent Psychiatry, Amsterdam University Medical Centre – Location VUMC, De Boelaan, Amsterdam, NL; 6 Youz: Parnassia Group, Dr. van Welylaan, The Hague, NL

**Keywords:** integrated care, families, parents, mental health, shared decision making

## Abstract

This article details a correction to the article: Nooteboom LA, Kuiper CHZ, Mulder E, Roetman PJ, Eilander J, Vermeiren RRJM. What Do Parents Expect in the 21st Century? A Qualitative Analysis of Integrated Youth Care. International Journal of Integrated Care. 2020; 20(3): 8. DOI: http://doi.org/10.5334/ijic.5419

## Correction

Nooteboom et al. (2020) was published with an incorrect citation provided. The original text cites:

“*A lack of access and availability negatively influences the care processes, for example by lowering attendance for appointments [21–52]*.” (p.8)

The citation at the end of this sentence should only cite references 21 and 52, not the full range. The correct sentence reads:

“*A lack of access and availability negatively influences the care processes, for example by lowering attendance for appointments[21, 52]*.”
