# Supplementary material for: What Do Parents Expect in the 21st Century? A Qualitative Analysis of Integrated Youth Care
Source: Int J Integr Care. 2020 Aug 17;20(3):8. doi: 10.5334/ijic.5419 (PMC7442175; doi:10.5334/ijic.5419)
Supplement: APPENDIX B. — Components and code scheme. [file ijic-20-3-5419-s2.pdf]

APPENDIX B: Components and code scheme

**Component**	**Description**	**Codes from code scheme**
Holistic, family-centered approach	A holistic approach of needs and strengths of all family members.	Family-centered focus<br>Broad focus on needs<br>Social Network
Address a broad range of needs in a timely manner	Timely support, tailored to a family's needs.	Timely signaling<br>Prevention<br>Access to care<br>Scale up/down<br>Visibility of professionals
Shared decision making	Parental involvement in decision making processes.	Shared care plan<br>Shared decision making<br>Freedom of choice<br>Point of view parent versus professional
Interprofessional collaboration	Collaboration between professionals with different expertise, or from different organizations.	Communication professionals, Collaboration professionals<br>Co-location<br>Coordination<br>Multidisciplinary meetings
Referral	Transition from one care provider/organization to another.	Referral<br>End of a care trajectory<br>Evaluation of a care process
Privacy	Privacy of family members during information exchange.	Privacy<br>Trust
